# Hospital Clinical Services and the New Age

**Published:** 1918-11-30

**Authors:** 


### HOSPITAL CLINICAL SERVICES AND THE NEW AGE.

The sense of impending change in the organisation and status of the medical profession has been canvassed principally in relation to the general practitioner, and naturally so, seeing that the great majority of the profession come under this designation. Inevitably, however, any considerable change affecting the activities of family practice would have some influence on the consultant branch of the profession, and it may be taken as certain that either by modification of the National Insurance Act or by some other means an arrangement will be made to secure for the industrial population the advantages of highly expert advice where the need for this exists. A defect in the present Insurance scheme in this respect is allowed, and opportunity will certainly be made to remedy it. Thus the interests of the consultant practitioner are involved in present discussions, and if hitherto he has dwelt on some serene height safe from popular agitation this immunity will certainly not continue. With rare exceptions he stood aloof from the agitation which disturbed the profession some few years ago, but in the next legislation dealing with medical politics he will find his position closely touched. Possibly then the importance of an active professional organisation will appeal to him.

In addition, however, to the effects of schemes involving more or less closely the general body of the profession there are signs that the special position of the consultant practitioner is coming under review, and this more particularly in relation to hospital practice. The demand for efficiency and thoroughness is beginning to ask whether the clinical service in our hospitals is at a level appropriate to the day and hour, and whether in this respect a considerable improvement is not possible. No one doubts that much excellent work is done in the wards or that the help given to the individual sufferer is valuable beyond words. Equally it is admitted that these results mean much gratuitous work, and even self-denial, from the hospital staffs. But it is remarked, and with justice, that the positions which these gentlemen hold give them great opportunities, and naturally the question arises whether these opportunities are used to the fullest advantage. They certainly ought to lead to considerable contributions to our knowledge of disease and to practical advances in relation both to prevention and to treatment. Here, again, it must be freely allowed that the record is far from being a blank one. British medicine can make great claims, and most of these, though by no means all, are the issue of hospital work and observation. Still, however, appears the question whether the event is equal to the occasion, and whether the conditions of our clinical services are such as to render possible the most fruitful work and to encourage this result. In passing judgment on such a question it must be remembered that the hospital staffs are honorary officers, and that their reward comes, if it comes at all, from the status which hospital appointments confer upon them. Such reward is always a postponed payment, and it is not infallibly secure. In such circumstances it is inevitable that more or less frequently hospital responsibilities must come into conflict with personal interests, and no one can expect that the latter should be neglected. Further, gratuitous work can hardly be reviewed with the severity which is commonly applied to recompensed duties, nor is it readily subject to censure or discipline. This principle exactly covers the present position of our hospital staffs, and on the whole it has worked not unsatisfactorily. Patients have been cared for, students have been trained, and physicians and surgeons have gained a large and valuable experience, and through all these results the interests of the general public have been served. The chapter of our voluntary hospitals has many satisfactory records and some brilliant ones, even when it is read solely from the point of view of a medical critic earnestly interested in the advance of the usefulness and capacity of his profession.

But when all the smooth things have been fairly said, it has still to be remarked that the good is not necessarily the best, and that the question which is appropriate to the spirit of the hour will certainly be applied to our hospital clinical services. The question, in other words, which this organisation, equally with many others, has to face is not, Does it work well, but Can it be made better? The answer may be in the affirmative without any general and unfair reflection on the past, but in any event we believe it will be in the affirmative. To take one aspect of the matter only, we are convinced that more time and attendance will be required from our hospital staffs. A common rule is to demand from each member attendance on two days in each week, and though in not a few instances more, and even much more, is given, this cannot be enforced, and the general level naturally is fixed by the formal demand. It is in the highest degree probable that this demand will be raised, even though such a decision should involve the disappearance of honorary service. More thorough and personal attention will thus be secured for patients, more sustained training for the student, and, still further, the staff will in this way accommodate itself to the modern conditions of exact observation and will be in a position to satisfy the demand that to "whomsoever much is given, of him shall be much required."

